# The Transcriptomic Landscape of Molecular Effects after Sublethal Exposure to Dinotefuran on *Apis mellifera*

**DOI:** 10.3390/insects12100898

**Published:** 2021-10-02

**Authors:** Yuhao Zhang, Yali Du, Weihua Ma, Jinjia Liu, Yusuo Jiang

**Affiliations:** College of Animal Sciences, Shanxi Agricultural University, Taigu 030801, China; zhangyuhao1005@126.com (Y.Z.); duyali2000@yeah.net (Y.D.); mawh1997@163.com (W.M.); liujinjia_819@163.com (J.L.)

**Keywords:** *Apis mellifera*, dinotefuran, transcriptome analysis, adverse impacts

## Abstract

**Simple Summary:**

*Apis mellifera* is one of the most important pollinator communities in nature. Insecticide residues in pollen and nectar, due to their wide use, may harm bees. Thus, it is crucial to provide novel insights into the effects of neonicotinoid insecticides on pollinators for protecting bees and maintaining a long-term stable ecological environment. The aim of our study was to investigate the effect and the mechanisms underlying bees impaired by dinotefuran. In the present study, for the first time, we found the mRNA expression profile of bees changes after treatment with sublethal doses of dinotefuran. Overall, our findings enhance understanding of the molecular mechanisms that underly physiological and behavioural damage for bees after dinotefuran exposure.

**Abstract:**

The decreasing number of bees is a global ecological problem. With the advancement of agricultural modernisation, the large-scale use of neonicotinoid insecticides is one of the main factors leading to the decline of bees. The aim of the present study was to investigate the effect and the mechanisms underlying bees impaired by dinotefuran. Acute (48 h) oral toxicity tests showed that a 5% lethal concentration (LC_5_) was 0.220 mg/L, and a 20% lethal concentration (LC_20_) was 0.458 mg/L. The gene expression profile shows that when compared with the control group, the LC_5_ group induced 206 significantly upregulated, differentially expressed genes (DEGs) and 363 significantly downregulated DEGs, while the LC_20_ group induced 180 significantly upregulated DEGs and 419 significantly downregulated DEGs. Significantly, transcriptomic analysis revealed DEGs involved in immunity, detoxification, and the nervous system, such as *antimicrobial peptides*, *vitellogenin*, *synaptotagmin-10*, *AChE-2*, and *nAChRa9*. Furthermore, Gene Ontology (GO) annotation and Kyoto Encyclopaedia of Genes and Genomes (KEGG) pathway analysis revealed that DEGs were enriched in amino acid and fatty acid biosynthesis and metabolism pathways. Collectively, our findings will help clarify the deleterious physiological and behavioural impacts of dinotefuran on bees and provide a basis for future research on the mechanisms underlying bees impaired by dinotefuran.

## 1. Introduction

Bees (Insecta: Hymenoptera: Apocrita: Apidae: *Apis*) are insects with apparent hierarchical differentiation and social division of labour [1] that provide humans with a wealth of bee products. There is emerging evidence that bees are the most abundant pollinator communities in nature; approximately 73% of crop pollination is performed by bees [2]. Moreover, bees are the only pollinating insects for most seed plants, and for many endangered plants, the germline is completed only after pollination by bees. Therefore, bees are crucial insects for protecting natural biodiversity and maintaining ecological balance [3]. Nevertheless, since the winter of 2006, colony collapse disorder (CCD) caused a large-scale loss of local bee colonies in the United States [4]. Farooqui systematically analysed various stress factors that caused the CCD phenomenon and believes that the abuse of chemical pesticides, especially the large-scale use of neonicotinoids, was closely related to the reduced numbers of bees [5]. Protecting bees and maintaining a long-term stable ecological environment is a major global challenge.

Neonicotinoid insecticides are a breakthrough after pyrethroid insecticides in the history of insecticides [6]. Since arriving on the market in the 1990s, neonicotinoid insecticides have been widely used to control piercing and sucking mouthpart pests, cotton aphids, leafhoppers, and planthopper pests [7]. However, bees need to engage in collection activities, and they are often exposed to harmful substances. Moreover, compared with other insects, the bee genome lacks genes encoding detoxification enzymes, making it more susceptible to neonicotinoid insecticides [8]. Previous studies have shown that bees prefer to collect nectar or pollen that contain neonicotinoid pesticides, which seriously endanger bees’ health and impair consumers’ health through bee products [9]. In recent years, research on the sublethal effects of neonicotinoid insecticides on honeybees has become a new hotspot. A large number of studies have shown that sublethal doses of neonicotinoid insecticides will seriously affect the development of a colony, including the development of the queen’s reproductive system, egg-laying behaviour, the growth and development of eggs, the lifespan, information exchange, navigation, positioning, collection, and flight of adult worker bees [10,11,12].

Dinotefuran is a third-generation neonicotinoid insecticide that binds to acetylcholine receptors in the insect neurotransmission system, disrupting neurotransmission and causing abnormal excitement, spasm paralysis, and death throughout the body [13]. Because dinotefuran has a characteristic structure, its repellent effect is better than that of traditional nicotinic insecticides. Studies have determined that under laboratory conditions, the acute toxicity of dinotefuran causes fatigue, low movement, struggle, falling, and kicking sideways in adult worker bees [14]. After treating newly entered worker bees with a LC_10_ dose of dinotefuran, the differentially expressed miRNAs in the brain are mainly related to development, nerve conduction, and immune defence [15]. The activities of acetylcholinesterase and polyphenol oxidase in the collection bees were significantly reduced after exposure to dinotefuran for 24 h [16]. A previous study found that S-dinotefuran’s acute oral toxicity to juvenile worker bees is higher than that of R-dinotefuran, while the inhibitory effect of R-dinotefuran on honeybees’ learning and memory ability was greater than that of S-dinotefuran, which may be associated with high expression of *SERCA*, *Kca,* and *Maxik* genes [17]. A recent study indicated that when young adult bees were exposed to dinotefuran, differentially expressed lncRNAs were mainly involved in development and the immune response [18]. However, to the best of our knowledge, there is no information about whether the mRNA expression profile of bees changes after treatment with a sublethal dose of dinotefuran. Therefore, the objective of the present study was to explore the detailed mode of dinotefuran action on *Apis mellifera* and determine which genes and metabolic pathways are affected by dinotefuran.

## 2. Materials and Methods

### 2.1. Honey Bees

*Apis mellifera* was artificially controlled and reared in the experimental apiary of Shanxi Agricultural University under natural outdoor conditions. At 9 o’clock in the morning, a total of 90 pollen foragers, respectively, from three healthy hives were collected using tweezers at the entrance of the hive [19]. Then placed them in plastic breeding boxes (8 × 8 × 6.5 cm^3^). After starvation for 2 h, treated bees with dinotefuran or 50% sterile sucrose solution (*w/w*), then placed cages in the incubator which were kept in darkness (28 °C, 65 ± 5% RH). Dinotefuran powder (MedChemExpress, Monmouth Junction, NJ, USA) was firstly dissolved in ddH_2_O then diluted to 50% sterile sucrose solution (*w*/*w*) to obtain the corresponding concentration dinotefuran.

### 2.2. Exposure to Sublethal Concentrations of Dinotefuran

Followed by starving for 2 h, different concentrations of dinotefuran sucrose solution (0.09375, 0.1875, 0.375, 0.75, 1.5, 3 mg/L) were added to the feeding boxes (15 µL/bee). Then, 50% sucrose solution (unlimited) was supplied after dinotefuran was consumed. During this period, we observed and removed dead bees. Finally, the number of dead bees was recorded after exposured to dinotefuran for 48 h. A 5% lethal concentration (LC_5_) and a 20% lethal concentration (LC_20_) were determined by SPSS Statistics 26 software [20].

According to the acute oral toxicity test results, LC_5_ of 0.220 mg/L and LC_20_ of 0.458 mg/L were selected as the experimental doses. The control group was fed 50% sucrose solution, and the treatment groups were fed corresponding sublethal concentration of dinotefuran. After dinotefuran was consumed, we supplied 50% sucrose solution for feeding. Three replicates were performed per sample. Checked breeding cages every day and removed dead bees. After 48 h, bee samples were quickly frozen in liquid nitrogen and then stored at −80 °C until used for RNA sequencing.

### 2.3. RNA Preparation and Library Construction

Total RNA was extracted from three whole bees of LC_5_ group, LC_20_ group, and control group, respectively, using TRIzol reagent (Invitrogen, Carlsbad, CA, USA) by following the supplier′s protocol. The concentration and purity of isolated total RNA were evaluated using a NanoDrop 2000 instrument (Nanodrop Instruments, Wilmington, DE, USA). mRNA was purified from total RNA using poly-T oligo-attached magnetic beads. The NEBNext^®^UltraTM RNA Library Prep Kit for Illumina^®^ (NEB, Ipswich, MA, USA) was used to construct the library, the Agilent Bioanalyzer 2100 system was used to assess the quality of the library and the effective concentration of the library. These libraries were paired-end (PE) sequenced based on the Illumina HiSeq sequencing platform. The RNA library construction was carried out by Beijing Biomark Biotechnology Co., Ltd. (Beijing, China) (http://www.biomarker.com.cn/, accessed on 4 March 2021).

### 2.4. RNA Sequencing and Analysis

Clean reads were filtered by removing the adaptor sequence and low-quality reads of raw data. Then, the clean reads were mapped to the *Apis mellifera* L. reference genome (Amel_4.5) to analyse expression and distribution on the genome. Differentially expressed genes were screened by |log2 Foldchange| > 1.5, *p* < 0.05. GO enrichment analysis of the DEGs was implemented by the GOseq R packages based on Wallenius noncentral hypergeometric distribution, which adjust for gene length bias DEGs. Statistical enrichment of DEGs in the KEGG pathway was tested using KOBAS software with *p* < 0.05.

### 2.5. Real-Time Quantitative PCR

Total RNA was extracted from three bees of each sample using TRIzol reagent. cDNA was synthesised using a reverse transcription kit (TAKARA Co., Ltd. Dalian, China). Quantitative real-time PCR (qPCR) was performed using the CFX qRT-PCR detection system (Bio-Rad, Hercules, CA, USA) and a SYBR Green qRT-PCR kit (TAKARA Co., Ltd. Dalian, China) according to the manufacturer′s instructions. Relative mRNA content was normalised to β-actin (GenBank accession number: NM_001185145.1) content, and the 2^−ΔΔCt^ method was used to determine relative changes in gene expression. The primer sequences are shown in Table S1.

### 2.6. Statistical Analysis

All data were analysed using the GraphPad Prism 7 software package (Monrovia, CA, USA). Data are expressed as the mean ± standard error of the mean (SEM). Student’s *t*-test with a two-tailed distribution was used, and *p* < 0.05 was considered statistically significant for all data.

## 3. Results

### 3.1. Acute (48 h) Oral Toxicity of Dinotefuran

**Figure 1:**
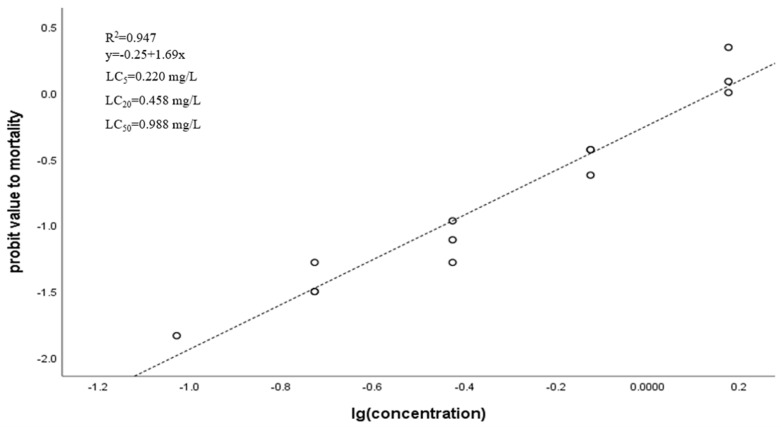
Regression lines of dinotefuran to honeybees.

During the experiment, compared with the control group, bees treated with low doses had no obvious poisoning symptoms. As the dose increased, the bees showed excitement and uncoordinated actions, began to roll, shake quickly, exhibited dyskinesia, continued to be excited and began to die. As shown in Figure 1, the LC_5_, LC_20_ and LC_50_ of dinotefuran were 0.22, 0.458, and 0.988 mg/L, respectively.

### 3.2. Mapping RNA-seq Reads to the Apis Mellifera Genome

RNA-seq obtained nine cDNA libraries representing control (CK-1, CK-2, CK-3), low-concentration treatment groups (LC_5_-1, LC_5_-2, LC_5_-3), and high-concentration treatment groups (LC_20_-1, LC_20_-2, LC_20_-3). The average clean reads of each group were 23,429,432, 21,311,514, and 20,256,909, respectively, and the percentage of Q30 bases of the three groups were all above 91.09% (Table 1). The raw sequence data were deposited in the NCBI Sequence Read Archive (SRA, accession number: SRP319973) and 80.89%~90.74% of the reads in our libraries were uniquely mapped to the reference genome (Amel_4.5) for the sequencing libraries.

### 3.3. Differential Gene Expression Analysis

**Figure 2:**
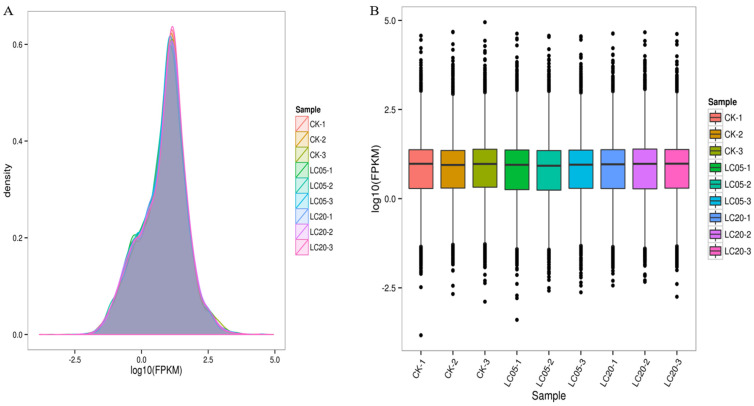
Bioinformatic analyses of RNA-seq data. (**A**) Density distribution of FPKM for each group; (**B**) FPKM density distribution boxplot for each group.

**Figure 3:**
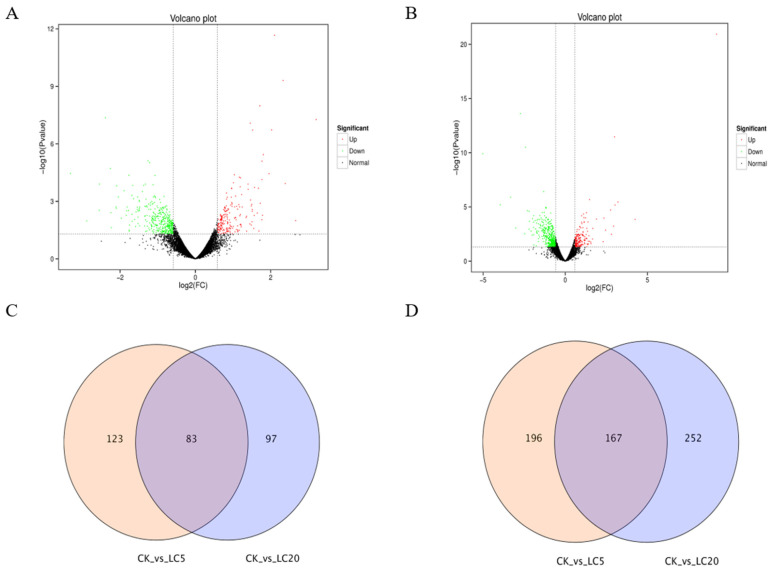
Bioinformatics analysis of differentially expressed genes (DEGs). (**A**) DEGs distribution of the control group (CK) and low concentration treatment group (LC_5_); (**B**) DEGs distribution of CK and high concentration treatment group (LC_20_); (**C**) Venn diagram of upregulated DEGs; (**D**) Venn diagram of downregulated DEGs.

As Figure 2 shows, fermented palm kernel meal (FPKM) and density distribution both revealed that most genes belong to the same group. DEGs, for example immune genes (*apidaecin, abaecin, hymenoptaecin*), the CYP450 family *(cyp304a1, cyp6a14, cypb561, cyp6a21, cyp6a17, cyp6b1, cyp4aa1*), leucine-rich repetitive sequence structure-like protein, and nicotinic acetylcholine receptor genes (*nAChRb2*, *nAChRa9*) were identified between different groups. Compared with the control group, the LC_5_ group included 206 significantly upregulated DEGs and 363 significantly downregulated DEGs (Figure 3A,C,D). The LC_20_ group had 180 significantly upregulated DEGs and 419 significantly downregulated DEGs when compared with control group (Figure 3B–D). Compared with the control group, there were 197 overlapping genes in the LC_5_ group and the LC_20_ group (Table S2).

### 3.4. Analysis of GO Enrichment and KEGG Enrichment of DEGs

**Figure 4:**
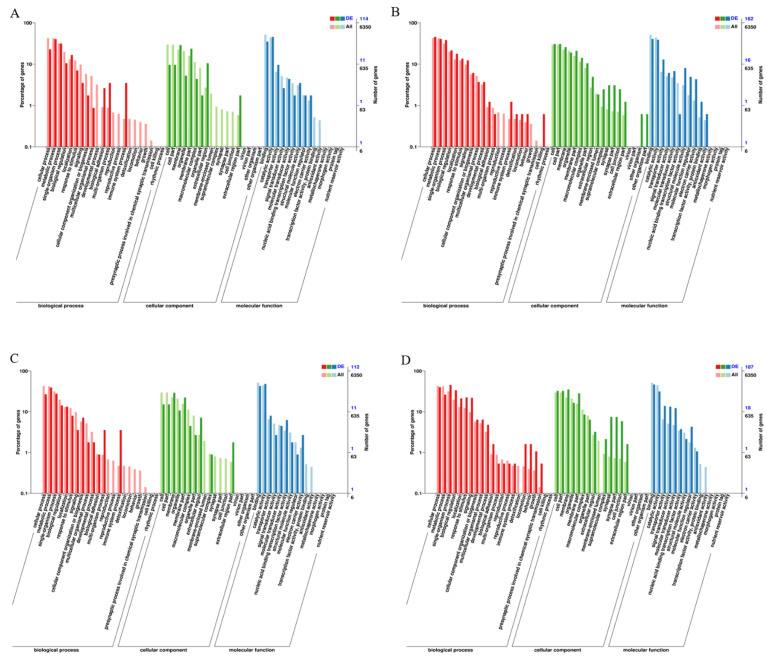

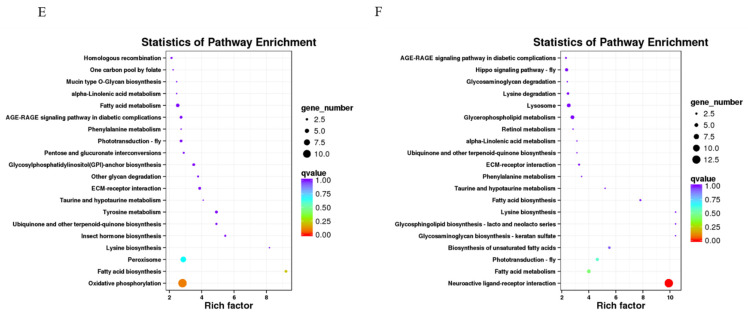
GO (Gene Ontology) enrichment analysis of differentially expressed genes (DEGs). (**A**) Upregulated DEGs distribution of the control group and LC_5_ group; (**B**) downregulated DEGs distribution of the control group and LC_5_ group; (**C**) upregulated DEGs distribution of the control group and LC_20_ group; (**D**) downregulated DEGs distribution of the control group and LC_20_ group; (**E**,**F**) KEGG enrichment analysis of DEGs; (**E**) DEGs distribution of the control group and LC_5_ group; (**F**) DEGs distribution of the control group and LC_20_ group.

To further understand the main functions of DEGs, we conducted GO analysis. The results showed that the upregulated genes of the LC_5_ group were primarily enriched in aromatic amino acid family metabolism, biosynthesis, bacterial defence, innate immune response, transport, fatty acyl-CoA reductase activity, and amino acid transmembrane transport activity (Figure 4A), while the downregulated genes were mainly enriched in GO classifications such as ion transport, skeletal myofibril assembly, potassium ion transmembrane transport, transiation, actin filament, postsynaptic membrane, cell junction, plasma membrane, potassium channel inhibitor activity, ubiquino-cytochrome-c reductase activity, cytochrome C oxidase activity and extracellular ligand-gated ion channel activity (Figure 4B). The upregulated genes of the LC_20_ group were primarily enriched in the oxidation–reduction process, transport, bacterial defence, innate immune response, the sphingomyelin catabolic process, phosphorylase kinase complex, glycerol-3-phosphate dehydrogenase complex, iron ion binding, monooxygenase activity, and oxidoreductase activity (Figure 4C), while the downregulated genes were mainly enriched in potassium ion transmembrane transport, ion transport, obsolete GTP catabolic process, postsynaptic membrane, acetylcholine-gated cation-selective channel activity, G-protein coupled GABA receptor activity, GABA-A receptor activity, ionotropic glutamate receptor activity, and extracellular-glutamate gated ion channel activity (Figure 4D). In general, the number of upregulated genes enriched in the GO classification related to defence mechanisms was greater than that of downregulated genes. The numbers of genes related to the redox process, immune response, and aromatic amino acid family metabolic process were 12, 7, and 3, respectively. As Figure 4E shows, most of the enriched KEGG pathways in the LC_5_ group were related to insect hormone biosynthesis, the MAPK pathway, FOXO signalling pathway, neuroactive ligand-receptor interaction pathway, and mTOR signalling pathway. For the LC_20_ group, the DEG-enriched KEGG pathway was involved in the interaction with the neural activity ligand-receptor, phenylalanine, fatty acid, purine, glycerophospholipid, taurine, and related metabolic signalling pathways (Figure 4F). These results further indicated that dinotefuran affected many vital metabolic pathways of honeybees.

### 3.5. Validation of DEGs by qPCR

**Figure 5:**
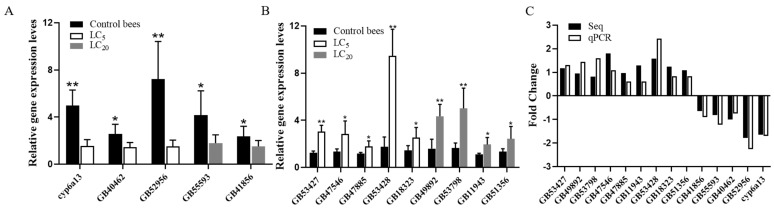
qPCR analysis of DEGs between dinotefuran-treated and control bees. (**A**) The relative expression levels of 5 randomly selected down-regulated DEGs between dinotefuran-treated bees and control bees; (**B**) the relative expression levels of nine randomly selected upregulated DEGs between dinotefuran-treated bees and control bees; (**C**) fold change of the dinotefuran-treated bees versus the control bees was verified by qPCR.

We verified the accuracy of transcriptome sequencing results by RT-qPCR of 14 DEGs. The relative expression levels of the five DEGs were significantly lower in dinotefuran-treated bees than control bees (*p* < 0.05; *p* < 0.01) (Figure 5A). The relative expression levels of the nine DEGs were significantly higher in dinotefuran-treated bees than control bees (*p* < 0.05; *p* < 0.01) (Figure 5B). All 14 selected DEGs were significantly differentially expressed in dinotefuran-treated bees compared with control bees (*p* < 0.05), and the DEGs expression fold changes were consistent with our RNA-seq data (Figure 5C).

## 4. Discussion

The large-scale use of dinotefuran caused residues in nectar and pollen of crops, which had tremendous negative impacts on bee populations. Therefore it is necessary to study the effects of sublethal doses of neonicotinoid insecticides on the health of insects. In the present study, LC_50_ values of dinotefuran tested at 48 h were 0.988 mg/L, which is similar to the previous study, indicating that dinotefuran has exceptional toxicity to adult *Apis mellifera* (oral LC_50_ = 1.29 mg/L) [16].

To further study the molecular mechanism causing honeybees’ death by dinotefuran, RNA-seq technology was employed to analyse and compare the differential gene expression between untreated control groups and the dinotefuran treated group. When compared with the control group, we found that 257 DEGs in LC_20_ group also appeared in the LC_5_ group. However, as the concentration increased, many DEGs were downregulated, indicating that a low concentration of dinotefuran may induce related genes to respond quickly. Similarly, exposure to sublethal imidacloprid reduced H_2_O_2_ production of common genes by inhibiting Duox gene transcription, leading to microbial regulatory capacity loss [21]. The neonicotinoid insecticide thiamethoxam negatively regulates the NF-kB immune signal in insects and negatively affects the antiviral defence of honeybees controlled by this transcription factor, thereby impairing immune defence and causing acute oral toxicity [22].

Studies showed that pesticides affect the detoxification and immune response of insects [23]. Our results indicated that bees may respond to dinotefuran by regulating key genes, metabolites, and signal transduction pathways. For example, in the present study, *vitellogenin* gene expression in dinotefuran treatment groups was upregulated, which plays a vital role in the immune response and longevity regulation of worker bees [24] and has a protective effect on insecticide-induced oxidative exposure [25], indicating it might play important roles after dinotefuran exposure to bees. Additionally, our study found that the immune genes *hymenoptaecin, abaecin*, and *apidaecins1* were significantly upregulated in the dinotefuran treatment groups, indicating that these three genes may be promising markers for monitoring bees’ response to dinotefuran exposure. The resistance of insects to insecticides mainly related to three major detoxification enzymes: cytochrome P450 monooxygenase (P450s), carboxylesterase (CarE), and glutathione S-transferase (GST) [26]. Recent studies indicated that *CYP6AS14* upregulated after bees exposed to the neonicotinoid insecticides thiacloprid and imidacloprid, suggesting it plays a vital role in the detoxification of these two neonicotinoid insecticides [27]. Similarly, RT-qPCR analysis confirmed that the transcription of *CYP6AS14, CYP304A1, CYP305A1, CYP6AS17*, and *CYP4g11* was upregulated in dinotefuran-treated groups.

GST provides protection against pyrethroid insecticides by combining with the molecules of pyrethroid insecticides in a chelating mechanism [28]. However, in the present study, there was no significant difference in GST expression after honeybees were exposed to dinotefuran. We speculated that GST does not play a significant role in the defence against dinotefuran. The AChE enzyme stops nerve impulses in the nervous system by catalysing acetylcholine hydrolysis, thereby playing a pivotal role in the nervous system [29]. As an agonist of the neurotransmitter acetylcholine, neonicotinoid compounds bind to insects’ nicotinic receptors, leading to long-term activation of the receptors until insects die [30]. Under the action of dinotefuran, the expression of AChE-2 gene in honeybees was significantly downregulated. All four neonicotinoid active ingredients had inhibitory effects on acetylcholinesterase activity [31]. The KEGG analysis results revealed that most of the DEGs were related to material metabolism and were mainly enriched in the fatty acid metabolism, amino acid metabolism, insect hormone synthesis, nucleic acid metabolism, and other metabolic pathways, similar to the results reported that differentially expressed lncRNAs in dinotefuran-treated groups enriched in carbohydrate and protein metabolism [18].

Low-dose neonicotinoid insecticides reduce Bombus foraging enthusiasm, slow foraging speed [32], and weaken the ability of bees to learn, smell, and remember [33]. OBP plays a vital role in the sensitivity and cognitive ability of the olfactory system [34]. OBP21 is expressed in bees’ glands that synthesise pheromones and participate in the synthesis, storage, and release of chemical pheromones [35]. Nicotinic acetylcholine receptors (nAChRs) are present in insect nerve tissue at high density and are targeted by neonicotinoid insecticides [36]. After excitotoxicity, the NPAS4–Syt10 signalling pathway plays an essential role in the neuronal response to solid synaptic activity [37]. We found that the expression of several genes related to smell, synapses, and pathways involved in the nervous system, such as *obp1, obp5, nAChRb2, nAChRa9, synaptotagmin-10,* and neuroactive ligand-receptor interactions, changed in honeybees affected by dinotefurcan. In addition, lipids are one of the primary sources of energy for organisms, but many lipids and their metabolites participate in various physiological metabolic processes. Studies have shown that the metabolism of the small molecule pheromone 2-heptanone, derived from the keto acid produced by lipase through the b-oxidation of long-chain fatty acids, is essential for collecting bees [38]. Additionally, 2-Heptanone is used as an alarm pheromone to enhance defence capabilities or as an odour marker to improve harvesting bees’ foraging efficiency [39]. Many DEGs related to lipid metabolism were found in this study, including fatty acid synthase, acyl-coenzyme, enoyl-coenzyme A, diacylglycerol kinase, phosphodiesterase, PLCB1, ultralong-chain fatty acid elongation protein, and fatty acid binding protein.

## 5. Conclusions

This study presented the first description of transcriptome expression profiling in bees affected by dinotefuran. In summation, dinotefuran interferes with the transcriptional and metabolic regulation network of honeybees. Honeybees strive to coordinate key defence pathways, such as redox processes, detoxification, and immune and energy metabolism, to maintain their survival ability under dinotefuran exposure. Our findings will improve understanding of the molecular mechanisms behind bee physiological and behavioural damage under dinotefuran exposure.

## Figures and Tables

**Table 1:** Sequencing data quality analysis and reference genome comparison results.

Simple Name	Clean Reads	Q30 (%)	Total Mapped	Mapped Reads	Unique Mapped Reads
CK-1	27,658,539	94.85%	55,317,078	50,192,862 (90.74%)	48,804,054 (88.23%)
CK-2	20,392,556	91.24%	40,785,112	33,658,507 (82.53%)	32,785,064 (80.38%)
CK-3	22,237,202	91.09%	44,474,404	38,962,393 (87.61%)	37,942,202 (85.31%)
LC05-1	20,561,037	94.72%	41,122,074	33,264,911 (80.89%)	32,254,135 (78.44%)
LC05-2	23,028,809	95.07%	46,057,618	37,855,789 (82.19%)	36,641,503 (79.56%)
LC05-3	20,344,696	94.87%	40,689,392	36,729,260 (90.27%)	35,098,847 (86.26%)
LC20-1	21,104,445	94.95%	42,208,890	37,238,021 (88.22%)	36,368,799 (86.16%)
LC20-2	19,639,136	95.09%	39,278,272	34,726,789 (88.41%)	34,104,678 (86.83%)
LC20-3	20,027,148	95.21%	40,054,296	35,496,091 (88.62%)	34,376,252 (85.82%)

## Data Availability

Data are contained within the article or supplementary material.

## Supplementary Materials

The following are available online at https://www.mdpi.com/article/10.3390/insects12100898/s1.
