# Supplementary material for: The Transcriptomic Landscape of Molecular Effects after Sublethal Exposure to Dinotefuran on Apis mellifera
Source: Insects. 2021 Oct 2;12(10):898. doi: 10.3390/insects12100898 (PMC8537135; doi:10.3390/insects12100898)
Supplement: Supplementary file 1 [file insects-12-00898-s001.zip › Table S1.pdf]

Table S1 Primer sequence

Name	Squence (5'-3')
β-actin	F: ATGCCAACACTGTCCTTTCTGG
	R: GACCCACCAATCCATACGGA
GB55593	F: ACGATTGTGACGTTTGTGGC
	R: TCACGGGAAGAAAAACTTTTCC
GB52956	F: TACCTGCTGTGCCAAGACAG
	R:CTCGTTGTACACCGGGAACA
Cyp4506a13	F: CGAGAAGTTGCCGCCAAATT
	R: CAAGTCGCCGAAATTCGCTT
GB40462	F: GGAAGGTTACCCTGGTGCTA
	R: ACTTCAGCAAGTGATAACGAAGAAC
GB41856	F: GACGCGAAGACCATATCCGT
	R: TCTGTGTCCTTGAAGTCCGC
GB53798	F: TCTTGTCCGAGCAACCGTAC
	R: GCAAACTCGTGTTCCGTGAC
GB49892	F: CGGTGGATCGCCTAATCTCA
	R: GGGTCAGCCCGTTCAGAAAT
GB11943	F: GTGGCGTTCAATCAACAGCA
	R: CCACCCGTGATATCGAAGCA
GB51356	F: CGTCGAGAAGATGCGCAAAG
	R: ACACCCATAGCGGTTTCCAG
GB47546	F: GGGTGAGGCGTGGGTATATC
	R: CGCTGTTTCTCTTCTTCTCTGC
GB18323	F: ATCTTCGCACTACTCGCCAC
	R: CTGACCAGGAAACGTTGGAA
GB53427	F: TGCGTACCATCGGTGAAACA
	R: ATCCGGCATCGATGAGTGTC
GB47885	F: TGTTCCCAGCTACATCTGCA
	R: ACGAGTCTTCCAGTTCCAACT
GB53428	F: GGGAACAGAGTCACCTCACG
	R: ATACCGACGCTTGTTACGCT
